# Preoperative Thyroid-Stimulating Hormone Levels and Risk of Thyroid Cancer in Post-thyroidectomy Patients for Thyroid Nodules: A Study From a Tertiary Hospital in Western Saudi Arabia

**DOI:** 10.7759/cureus.47622

**Published:** 2023-10-25

**Authors:** Hadi Afandi Al-Hakami, Jamelah F Altayyeb, Salwan M Alsharif, Mohammad A Alshareef, Baraa I Awad, Mohammed Al-Garni

**Affiliations:** 1 Otolaryngology - Head and Neck Surgery, King Saud Bin Abdulaziz University for Health Sciences, King Abdullah International Medical Research Center, Ministry of the National Guard Health Affairs, Jeddah, SAU; 2 College of Medicine, King Saud Bin Abdulaziz University for Health Sciences, Jeddah, SAU; 3 Otolaryngology - Head and Neck Surgery, King Saud Bin Abdulaziz University for Health Sciences, King Abdullah International Medical Research Center, Ministry of National Guard Health Affairs, Jeddah, SAU

**Keywords:** thyroid-stimulating hormone, preoperative tsh, differentiated thyroid cancer, tsh levels, thyroid nodules

## Abstract

Background

Although serum thyroid-stimulating hormone (TSH) is one of the basic investigations to assess thyroid nodules, its role in thyroid oncogenesis remains unclear. Previous literature has conflicting findings regarding TSH levels and the prediction of malignancy. This study aims to investigate the association between TSH levels and the risk of malignancy and advanced staging in patients who underwent thyroidectomy for nodular thyroid disease. Additionally, it aims to assess if higher TSH correlates with malignancy in Bethesda staging III, IV, and V.

Methodology

This retrospective cohort study was conducted among participants who underwent near-total/total thyroidectomy or hemithyroidectomy at King Abdulaziz Medical City between 2016 and 2021.

Results

A total of 378 cases were included, and 50.3% of the cases had malignant nodules in the surgical histopathology findings. The median TSH levels were higher in malignant nodules compared to benign ones (1.64 mIU/L versus 1.49 mIU/L; p < 0.001). Additionally, higher TSH levels were not associated with advanced staging or malignancy in patients with Bethesda stage III-V.

Conclusions

Higher TSH levels are associated with an increased risk of malignancy in patients with nodular thyroid disease. Using TSH levels as an adjunctive tool for identifying high-risk patients with thyroid nodules would aid in management planning.

## Introduction

A thyroid nodule, as defined by the American Thyroid Association (ATA), is “a discrete lesion within the thyroid gland that is radiologically distinct from the surrounding thyroid parenchyma” [1]. In general, thyroid nodules are a common condition seen in clinical practice. The prevalence varies based on the modality of detection. Clinically, 2-6% of the population have palpable nodules and 10-41% have thyroid nodules detected by ultrasonography [2,3]. As 7-15% of patients with thyroid nodules have malignant tumors, it is crucial to be able to determine suspicious cases among all patients with nodules [4]. Features that raise suspicion include having a personal history of head and neck radiation, young age (<20 years) or advanced age (>70 years), being a male, or having a family history of multiple endocrine neoplasia (MEN), medullary thyroid cancer (MTC), and papillary thyroid cancer (PTC) [1].

Globally, a sustained increase in thyroid cancer cases has been reported [5,6]. The Global Cancer Statistics 2020 (GLOBOCAN) estimated that 586,202 patients were diagnosed with thyroid cancer worldwide, with the highest rates in Eastern Asia (59.7%), Europe (14.9%), and Africa (10.8%) [7]. Over the last decades, there has been an increase in reporting the incidence of thyroid cancer in Saudi Arabia. Based on the latest systematic review published in 2020 by Alqahtani et al., the prevalence of thyroid cancer in Saudi Arabia is estimated to be 12.9% [8].

Ultrasound-guided fine-needle aspiration biopsy (FNAB-US) is the gold standard test to diagnose thyroid cancer [2,9]. In current practice, FNAB-US is indicated for >1 cm thyroid nodules or for <1 cm nodules with high-risk US features [1,10]. However, even with a high sensitivity and specificity of FNAB-US, there is a known possibility of inconclusive/indeterminate results [9,10]. Thus, the incorporation of the patient’s clinical profile, laboratory results, imaging, and FNAB results is the best approach for a definitive diagnosis of thyroid cancer and to decide a proper management plan [11].

In thyroid nodules, ATA guidelines strongly recommend ordering serum thyroid-stimulating hormone (TSH) as an initial step of evaluation and identification of thyroid dysfunction requiring treatment [11]. If TSH levels are suboptimal, the next step is to order scintigraphic imaging to determine the functionality of the nodules. Usually, the suboptimal levels of TSH may indicate a hyperfunctioning thyroid nodule which has a low risk of cancer [4]. Although serum TSH is one of the basic clinical investigations to assess thyroid nodules, it is not readily used in determining their malignancy risk. The relationship between TSH levels and thyroid cancer has been investigated in many studies. Previous literature found an association between higher serum TSH levels and an increase in the risk of differentiated thyroid cancer in patients with thyroid nodules [12,13]. These findings are not consistent with other studies that found a controversial role of serum TSH levels and the prediction of malignancy [14].

This study aims to investigate the association between TSH levels and the risk of malignancy and advanced staging in patients who underwent a thyroidectomy for nodular thyroid disease. Additionally, it aims to assess if higher TSH correlates with malignancy in Bethesda staging III, IV, and V.

## Materials and methods

A retrospective cohort study was conducted between May 2016 and December 2021 in King Abdulaziz Medical City (KAMC), a tertiary hospital in the western region of Saudi Arabia, Jeddah. The study subjects were patients under the care of the Department of Otolaryngology-Head and Neck Surgery. This study was approved by the Institutional Review Board at King Abdullah International Medical Research Center (reference number: JED-22-427780-118). Patient data were secured, held anonymously, and used only for the study purpose.

The included study subjects were all adult patients (>18 years) diagnosed with thyroid nodules by ultrasound who underwent near-total/total thyroidectomy or hemithyroidectomy. We excluded participants who were pediatric patients (<18 years), patients diagnosed with thyroid diseases that are known to cause either hyperthyroidism or hypothyroidism (e.g., Grave’s disease, Hashimoto thyroiditis, and congenital hypothyroidism), and patients on levothyroxine treatment.

Based on our study population, the minimum estimated sample size was 235 at a 95% confidence interval, 5% margin of error, and 80% study power. A convenient non-probability sampling technique was applied to select the required sample size among all patients who underwent thyroidectomy for thyroid nodules.

The data were collected from patients’ electronic medical records obtained from the BESTCare system at KAMC. A self-developed data collection sheet was used to collect and organize the recruited data. Continuous variables included age at surgical intervention, nodular size based on the last US (cm) before surgery, and last preoperative serum TSH levels (mIU/L). The categorical variables were gender, number of nodules seen on US, type of surgical intervention, final surgical histopathology diagnosis (benign versus malignant), type of malignancy (papillary, follicular, or undifferentiated thyroid cancer), staging of malignant cases following the American Joint Committee on Cancer (AJCC) eighth edition [15], and FNAB-US results, which were categorized using the Bethesda System (a standardized reporting system of FNA results). It is categorized as Bethesda I (non-diagnostic/unsatisfactory), Bethesda II (benign), Bethesda III (atypia of undetermined significance or follicular lesion of undetermined significance), Bethesda IV (follicular neoplasm or suspicious for follicular neoplasm), Bethesda V (suspicious for malignancy), and Bethesda VI (malignant) [16].

After collecting the data, they were entered and analyzed using SPSS Statistics for MacOS, version 27 (IBM Corp., Armonk, NY, USA). All categorical variables were summarized as frequencies and percentages. Skewed distribution numerical variables were presented as median and interquartile range values. For bivariate analyses, the chi-square test was used to analyze categorical data. The Spearman’s test was used to detect the correlation between numerical and ordinal variables. The binary logistic regression was used to predict malignancy. A p-value <0.05 was considered statistically significant.

## Results

A total of 378 cases were included in the study. The participants’ median age was 43 years with an interquartile range of 24 years. Overall, 77.2% of the cases were females, and the majority (73.5%) underwent near-total/total thyroidectomy. Details of demographic data, US nodular size, TSH levels, FNA results, type of thyroid surgery, and final surgical histopathology results are illustrated in Table 1.

**Table 1:** Demographic data and nodular characteristics.

Demographic data	N = 378	N	%
Age (years)	<45	199	52.6%
	45–55	80	21.2%
	>55	99	26.2%
Gender	Male	86	22.8%
	Female	292	77.2%
Type of thyroidectomy	Near-total/Total	278	73.5%
	Hemithyroidectomy	100	26.5%
Thyroid-stimulating levels (mIU/L)	<0.4	40	10.6%
	0.4–1.39	120	31.7%
	1.4–2.49	132	34.9%
	2.5–5	67	17.7%
	>5.0	19	5%
Nodular characteristics			
Size of nodules (cm)	<1	20	5.3%
	≥1	358	94.7%
Fine-needle aspiration results per Bethesda classification system	I	5	1.3%
	II	181	47.9%
	III	32	8.5%
	IV	39	10.3%
	V	27	7.1%
	VI	94	24.9%
Final surgical histopathological diagnosis	Benign nodules	188	49.7%
	Papillary thyroid carcinoma	181	47.9%
	Follicular thyroid cancer	5	1.3%
	Hurthle cell carcinoma	2	0.5%
	Undifferentiated carcinoma	2	0.5%

Based on the final surgical histopathology results, 188 (49.8%) of the cases were benign nodules, whereas 190 (50.3%) were malignant nodules. Most of the cases accounted for PTC 181 (95.3%). Furthermore, out of all 190 malignant cases, 78.4% were classified as stage I, followed by stage II (16.8%), and the rest were stage III (1.6%) and stage IV (3.2%).

We evaluated the correlation between malignancy of differentiated thyroid cancer and associated factors such as age, gender, and TSH levels (Table 2). Overall, patients with malignancies had higher median TSH levels than patients with benign nodules (1.49 mIU/L versus 1.64 mIU/L). This association achieved statistical significance (p < 0.001). Interestingly, both genders had almost an equal risk of malignancy, even though females were more than 70% of the cases in the study. This finding did not reach statistical significance (p = 0.955). Similarly, no significant association between age and malignancy was found (p = 0.229).

**Table 2:** The correlation between malignancy status and associated factors. IQR: interquartile range; *: chi-square; **: multiple logistic regression

Associated factors		Malignancy status				P-value
		Benign		Malignant		
		N	%	N	%	
Age	<45	91	45.7%	108	54.3%	0.229*
	45–55	45	56.3%	35	43.8%	
	>55	52	52.5%	47	47.5%	
Gender	Male	43	50.0%	43	50.0%	0.955*
	Female	145	49.7%	147	50.3%	
Thyroid-stimulating hormone levels	<0.4	23	57.5%	17	42.5%	0.025*
	0.4–1.39	66	55.0%	54	45.0%	
	1.4–2.49	68	51.5%	64	48.5%	
	2.5–5	27	40.3%	40	59.7%	
	>5.0	4	21.1%	15	78.9%	
	Median (IQR)	1.49 (1.31)		1.64 (1.64)		<0.001**

## Discussion

The clinical importance of thyroid nodules rests with the need to exclude thyroid cancer, which occurs in 7-15% of cases depending on age, sex, radiation exposure history, family history, and other factors [4]. Currently, however, it is not entirely clear whether TSH plays a role in cancer development, cancer progression, or both. It was suggested that the role of TSH in thyroid tissue is to promote cell proliferation and possible oncogene activation. This is supported by the fact that inducing pharmacological hyperthyroidism in patients with thyroid cancer will result in endogenous TSH suppression and subsequent inhibition of nodular growth [14,17]. Higher TSH levels as an independent predictor for differentiated thyroid cancer were examined in multiple studies [18-21]. In a study by Haymart et al., 843 cases were included and 241 were found to have differentiated thyroid cancer. Mean TSH was significantly higher in malignant pathology than in benign (2.5 mIU/L ± 0.3 vs. 1.6 mIU/L ± 0.1). The authors also observed an escalating risk of malignancy that is proportionate with an increase in TSH levels [18].

In our study, we demonstrated that preoperative TSH levels, even in normal ranges, were higher in patients with differentiated thyroid cancer compared to patients with non-malignant nodular thyroid disease regardless of age, gender, or nodular size. This is consistent with the recent meta-analysis of Su et al., which included 23,700 subjects from Chinese and non-Chinese populations. Their analysis showed a significant relationship between preoperative TSH levels and differentiated thyroid cancer. Each 1 mIU/L increase in TSH was associated with a 16% risk of having differentiated thyroid cancer in a non-Chinese population while a 25% risk in a Chinese population [19].

In contrast to our observation, the EPIC cohort study confirmed a negative association between pre-diagnostic TSH levels and malignancy risk (a median TSH level of 1.15 mIU/L compared to 1.30 mIU/L in the control group) [20]. A possible explanation for this controversy is that they were comparing TSH levels in patients with thyroid cancer with healthy individuals rather than patients with thyroid nodules.

Contrary to Haymart et al. [18] who reported that higher TSH levels were associated with advanced differentiated thyroid cancer staging, our study found no significant correlation between higher serum TSH and advanced staging in cases of differentiated thyroid cancer. Despite that, there was a non-significant trend for higher median TSH in stages III and IV compared to stages I and II (1.76 versus 1.69). This trend was also observed by Tam et al. [22] who found a similar non-significant elevation in serum TSH with advanced staging. They explained their conclusion by having only 1.3% of their cases in advanced stages. Our findings support the observations of Kim et al. [23] who also did not find an association between higher TSH and advanced tumor staging. They explained the contrast from previous studies by having a lower sample size and loss of follow-up. Part of their explanation could be applied to our study as there was a lack of follow-up after surgery. This obstructed the further assessment of nodular involvement and distant metastasis in some cases (Nx and Mx) in the surgical pathology report. Additionally, both Batool et al. and Soleimanisardoo et al. concur with our finding, suggesting that the escalating risk of disease progression may not be supported by serum TSH elevation [24,25].

As of now, the malignancy potential in indeterminate FNA categories (Bethesda III and IV) is variable in different institutes [8,26]. Moreover, other than surgical resection of the thyroid gland, there are no definitive measures to determine that thyroid nodules categorized as Bethesda III-V have indeed malignant etiologies rather than benign nodules [27]. In our study, we attempt to investigate the relationship between higher TSH levels in these groups and the risk of malignancy. An observation of a slight increase in TSH levels with each category that proved to be malignant was noted. However, no statistical significance was found regarding higher TSH levels in Bethesda III, IV, and V in malignant cases compared to benign ones. Thus, based on these results, there is no support for using TSH as a parameter for predicting malignancy in patients with Bethesda III-V. Likewise, Kuzu et al. studied the value of different blood cell markers as an indicator for malignancy in patients with Bethesda III. A total of 101 cases were included in the study, and 34 of them had malignancy at the final pathological diagnosis. Upon assessment of mean TSH, there was no correlation between the TSH levels and malignant thyroid nodules [27]. These observations were consistent with other studies [28-30].

The current study has a few limitations. First, the preoperative TSH measures were not collected in the same time frame in all cases. This could possibly interfere with establishing the correlation at the time of diagnosis. Another limitation is that the retrospective aspect of this study was targeted toward thyroidectomy patients with nodular disease. In turn, this narrows the window of applying the result over the nodular thyroid population who did not undergo surgery. Another gap could be the difficulty recognizing the cut-off value of TSH by receiver operating characteristic curve analysis that might serve as a predictor of cancer.

Studying the relationship between TSH and malignancy was not meant to be used as a sole diagnostic marker for the final treatment plan in patients with nodular thyroid disease. Using TSH levels as an adjuvant assessment tool, along with US findings, cytological findings, and patient characteristics, in patients with thyroid nodules aids in classifying these patients into either high-risk or low-risk groups. This would also help in reducing the number of unnecessary thyroidectomy procedures and limiting the surgical intervention only to those who have nodules with a high likelihood of malignancy. This reduces the surgical subsequent consequences and complications and overall improves the management decision-making (total thyroidectomy/hemithyroidectomy versus watchful waiting), especially in patients with uncertain diagnoses, which will eventually improve patient care.

## Conclusions

Our study supports the association between higher TSH levels, even within normal ranges, with an increased risk of having differentiated thyroid cancer in patients with nodular thyroid disease. Our findings suggest a role of TSH in the development of cancer with a poor understanding of the exact mechanism. In addition, there was no role for TSH in determining high-risk patients with Bethesda stage III-V in their FNA report. We suggest that preoperative serum TSH levels could be used as an adjuvant tool, rather than a sole test, in identifying high-risk patients with nodular thyroid disease to help decide the choice of near-total/total thyroidectomy, hemithyroidectomy, or watchful waiting, especially in uncertain diagnoses.
